# Resilience Among Healthcare Staff: A Randomized Controlled Trial of a Digital Training Program

**DOI:** 10.1007/s10880-025-10085-1

**Published:** 2025-06-22

**Authors:** Lotte Bock, Lara Westemeyer, Nadine Moschner, Majeed Rana, Madiha Rana

**Affiliations:** 1 Leuphana University of Lüneburg, Lüneburg, Germany; 2 Heinrich Heine University Düsseldorf, Düsseldorf, Germany; 3 Europäische Fernhochschule Hamburg, Hamburg, Germany

**Keywords:** Resilience, Nursing care staff, Online training programme

## Abstract

Nursing and direct care staff face significant psychological and physical challenges, including high workloads, emotional labour, and staff shortages, which increase the risk of burnout and stress-related conditions. Resilience is a protective factor that mitigates these effects. This study evaluated the efficacy of a 28-day digital resilience training programme tailored to the needs of nursing and direct care staff. One hundred twenty participants working in German healthcare settings were randomly assigned to an experimental group or a waitlist control group. Experimental group participants received a programme consisting of twice-daily video-based exercises, reflections, and relaxation activities over four weeks. Outcomes were assessed at baseline (T1), post-intervention (T2), and three months follow-up (T3). Resilience (primary outcome) was measured using the Resilience Scale, and perceived stress (secondary outcome) was measured using the Perceived Stress Questionnaire (PSQ). Compared to the control group, experimental group participants demonstrated significant improvements in resilience from T1 to T3 (*p* < .001, *d* = 0.6) and substantial reductions in percieved stress (PSQ total score, *p* < .001, *d* = − 1.4). No significant changes were observed in the control group. The digital programme effectively enhanced resilience and reduced the perception of stress, aligning with prior research on resilience training in healthcare. Despite stress reduction showing a larger effect size, resilience remained the theoretically grounded primary outcome. The flexible, scalable design of the digital programme accommodates challenges like shift work, offering a practical solution for improving nurse staff’s mental health. Clinical Trial Registration: German Clinical Trials Register (DRKS), Identifier: DRKS00030973.

## Introduction

Nursing and direct care staff are subject to considerable physical and mental challenges in their daily professional activities. The most common stressors include time pressure, staff shortages, shift work, and frequent exposure to death and suffering. These factors increase the risk of burnout, depression, and anxiety disorders (Breinbauer et al., 2019; Drupp & Meyer, 2019). The prevalence of psychological diagnoses among sick days indicates that these stressors are significant contributors to healthcare worker well-being. A significant contributing factor to this stress is emotional labour, which involves the management of one’s own and others’ emotions. In particular, "surface acting," or the adaptation of emotions to professional requirements, frequently results in emotional dissonance and, in the long term, increases the risk of burnout (Delgado et al., 2017). A substantial proportion of nursing and direct care staff report a sense of psychological emptiness and inner imbalance, a sentiment that is further exacerbated by the emotional demands of their profession (Hart et al., 2014). While experienced nursing and direct care staff encounter significant emotional and psychological burdens, nursing students also experience elevated levels of stress due to academic pressures, clinical responsibilities, and emotional challenges (Labrague et al., 2017).

Research indicates that resilience plays a pivotal role in enabling individuals to manage stress effectively (Li & Hasson, 2020). Conceptually, resilience refers to the capacity to adapt positively to adversity and to demonstrate psychological growth in the face of challenges. While traditionally rooted in developmental psychology, contemporary perspectives underscore its dynamic nature, shaped by individual, social, and contextual factors (Caldeira & Timmins, 2016). Within the nursing profession, resilience is closely linked to mental well-being and professional sustainability. Empirical studies have demonstrated that resilience mitigates the adverse effects of emotional labour and is inversely associated with burnout, emotional exhaustion, and depersonalization (Castillo-González et al., 2024; Delgado et al., 2017). Furthermore, higher resilience levels correlate with increased job satisfaction and reduced intent to leave the profession (Füreder, Herber & Stadlmayr, 2024; Manomenidis et al., 2019), as well as lower symptoms of post-traumatic stress and depression (Mealer et al., 2012b). Importantly, resilience is not an innate trait but a modifiable capacity that can be cultivated through targeted, evidence-based interventions. Strategies such as cognitive restructuring, mindfulness training, stress inoculation, and emotional regulation have demonstrated efficacy in enhancing psychological resilience among healthcare professionals (Arrogante & Aparicio-Zaldivar, 2017; Joyce et al., 2018; Mealer et al., 2012a, 2012b; Smith & Yang, 2017). These findings underscore the importance of systematically integrating resilience-building interventions into nursing education and professional development frameworks to foster enduring coping capacities.

While traditional face-to-face interventions have demonstrated effectiveness in enhancing resilience, the applicability of digital resilience training among nursing and direct care staff is still emerging. Existing digital programs vary widely in their delivery modes, duration, and target populations. For example, Zhai et al. (2021) conducted a meta-analysis that confirmed significant effects of resilience training interventions—including digital formats—on reducing stress and burnout in healthcare workers. However, the majority of studies included were not specifically tailored to nursing and direct care staff. The most evidence-supported resilience training strategies include cognitive-behavioral techniques, mindfulness-based interventions, and stress management skills training. These approaches have demonstrated adaptability across various formats and are particularly well-suited for further adaptation and implementation in the nursing context.

Despite these promising findings in broader healthcare contexts, there remains a clear research gap regarding the effectiveness of fully asynchronous, self-guided digital resilience interventions tailored specifically to the nursing and direct care staff. Most available programs do not accommodate the unique demands of shift work and are not tested with long-term follow-ups. Moreover, few interventions have been rigorously evaluated using randomized controlled designs.

As asserted by Thomas and Asselin (2018), Thomas and Revell (2015), and Amsrud et al. (2019), strengthening of resilience through targeted interventions plays a crucial role in clinical environments. Recent studies by Li and Hasson (2020) and Diffley and Duddle (2022) indicate that multicomponent interventions—including information sharing and reflective learning—are particularly effective in promoting resilience during nursing training. For working nursing and direct care staff, resilience has been shown to reduce emotional exhaustion and absenteeism, and to improve job satisfaction and performance (Matzka, 2015; Walpita & Arambepola, 2020). Furthermore, the implementation of resilience programs has been demonstrated to fortify professional identity and contribute to employee retention (Füreder, Herber & Stadlmayr, 2024).

In summary, the evidence underscores the importance of fostering resilience to safeguard the long-term mental health and performance of healthcare workers. Nurses and other direct care staff may benefit from the implementation of structured resilience training at all career stages to ensure preparation for the emotional and psychological demands of their roles. However, it is imperative that these interventions are both intensive and comprehensive in nature if they are to yield the desired outcomes.

### Research Question

The present study was predicated on the assumption that targeted resilience promotion can engender sustainable improvements in the mental health and well-being of nursing and direct care staff. To address a gap in the literature, we evaluated a 28-day self-paced, fully digital resilience training program designed specifically for nursing and direct care staff. Grounded in validated psychological models, the training delivers content asynchronously to accommodate variable schedules and workplace demands. Evidence-based resilience interventions often incorporate cognitive-behavioral strategies, mindfulness techniques, stress inoculation training, and emotional regulation—approaches that have shown efficacy in reducing burnout and enhancing well-being in healthcare settings. These established methods inform the program’s content and delivery. By using a randomized controlled design with three assessment points—including a three-month follow-up—we aimed to generate robust evidence regarding the program’s long-term effectiveness and feasibility in real-world healthcare contexts. The integration of psychological theories into the development and evaluation of the training programme ensures that scientifically sound and practice-relevant interventions are provided. The central hypotheses guiding this study were as follows::

#### Hypothesis 1

Nursing and direct care staff who have completed the online training will achieve higher scores on the Resistance Orientation–Regeneration Orientation Scale than staff in the waitlist control group.

#### Hypothesis 2

Nursing and direct care staff who have completed the online training will achieve lower scores on the Perceived Stress Questionnaire than staff in the waitlist control group.

## Methods

### Ethical Approval

Approval for this study was granted by the Euro-FH Ethics Committee (EKEFH07/24), and all participants provided written informed consent. All participants were fully informed of their right to withdraw at any time without consequence, that their participation was entirely voluntary, and that their decision to take part—or not—would have no impact on any aspect of their employment. No employer contact or institutional pressure was involved, and participants were not employed in the same organization as the investigators.

### Design

The randomised controlled trial was conducted over a period of three assessment points: baseline (T1), post-intervention (T2), and follow-up (T3). The study was conducted between July and November 2024. The experimental group commenced the 28-day online programme immediately after completing the baseline questionnaire (T1). In contrast, the waitlist control group did not receive access to the intervention during the study period and only began the training after completing the final follow-up assessment (T3). This waitlist design was selected to preserve the ability to compare both groups across all time points while ethically ensuring that all participants eventually had access to the programme. The final follow-up questionnaire (T3) was administered to all participants three months post-intervention. The study achieved a 100% retention rate, with all 120 participants completing all three assessments. This unusually high adherence was facilitated by sustained and proactive communication throughout the study period. One member of the research team maintained regular, personalised contact with participants, which served to clarify procedures, respond to technical issues, and encourage ongoing engagement. Although no monetary or material incentives were offered, this attentive support likely contributed to the completion rate.

### Participants

The required sample size (*N* = 120) was determined using G*Power 3.1 for an ANOVA repeated measures analysis (effect size *d* = 0.3, *α* = 0.05, *β* = 0.95). The effect size of *d* = 0.3 was chosen as it reflects a small-to-medium effect based on Cohen’s conventions (Cohen, 2013) and is consistent with prior meta-analyses of psychological resilience interventions in healthcare professionals, which have reported effect sizes ranging from *d* = 0.2–0.5 (Joyce et al., 2018; Zhai et al., 2021). This conservative estimate ensured sufficient power to detect realistic intervention effects in a real-world setting. Participants were recruited through direct outreach and were aged between 18 and 69 years (*M* = 51). They were employed in German healthcare settings. The final sample comprised 51 males and 69 females with diverse professional qualifications: 65 certified nurses, 13 nursing assistants, 28 individuals in training or without formal qualifications, 8 medical assistants, and 6 professionals in disability care roles. For the purpose of this study, the term “nursing and direct care staff” is used to describe all individuals actively engaged in direct patient or client care within healthcare settings, regardless of formal credentials. Although their qualifications and roles differ, these individuals commonly work in high-pressure, shift-based environments and are routinely exposed to emotional and physical stressors, making them an appropriate and relevant population for a resilience-focused intervention. The participants’ professional experience ranged from fewer than 10 years (*n* = 39) to more than 20 years (*n* = 40). The employment settings of the participants included acute care (*n* = 25), residential long-term care (*n* = 76), and outpatient care (*n* = 19). Most participants (*n* = 98) were engaged in full-time employment, while 20 were employed part-time and 2 held temporary employment.

Participants agreed to allocate 20 min per day to the resilience training programme. Adherence to the intervention was critical for the assessment of its effectiveness, and data collection was conducted through three completed questionnaires over the testing period. An online randomization tool was used to assign participants to either the experimental group or the waitlist control group. Prior to the baseline assessment (T1) and group allocation, all participants received comprehensive information regarding the study design and timeline.

### Intervention

The programme was informed by established stress theories, including Lazarus and Folkman’s (1986) transactional model of stress, as well as contemporary research on resilience and the effectiveness of online interventions (Ang et al., 2022; Chmitorz et al., 2018; Diaz et al., 2021; Fischer & Law, 2021). Over a 28-day period, participants in the experimental group received two daily emails—one in the morning at 7:00 am and another in the evening—designed to integrate seamlessly into their daily routines. The morning emails contained concise, video-based “learning nuggets” on resilience, supplemented with practical exercises and relaxation techniques such as breathing exercises, guided meditation, and simplified yoga (Hoffman, 2004; Morgan et al., 2024). Conversely, the evening emails promoted reflective practices, prompting participants to engage in journaling about positive daily experiences, gratitude, or meaningful social connections (Clark et al., 1995; Thompson, 1991; Ellis, 2023). Furthermore, an introductory video provided guidance on adapting the programme to individual schedules, thereby enhancing its accessibility and flexibility. Participants were encouraged to engage in approximately 20 min of content daily over the 28-day period. This duration was selected based on prior digital mental health interventions demonstrating efficacy with brief but consistent daily engagement (e.g., Ang et al., 2022; Bock et al., 2024). However, participation was not strictly enforced. The programme was designed to allow users to pause, resume, or combine sessions according to their schedules. This flexible design aimed to accommodate the realities of shift work, particularly in nursing and direct care staff. Adherence was self-paced and not externally monitored beyond completion of pre-, post-, and follow-up assessments.

### Programme Content by Week

The training programme was meticulously designed over a period of four weeks, with each week focusing on specific psychological principles and practices to enhance resilience.

The programme commenced with an inaugural week, the objective of which was to establish a foundation of understanding and familiarity with the principles and practices of resilience. The programme commenced with an introduction to the fundamental principles of resilience, facilitated through daily reflection routines and relaxation exercises. Key topics included neuropsychological mechanisms, the role of sleep in emotional well-being, personal resource activation, and emotional regulation (Hoffman, 2004; Morgan et al., 2024). In the subsequent week, participants were guided through a series of exercises aimed at fostering a mindset of acceptance and equipping them with coping mechanisms. Participants were guided in cultivating a solution-oriented mindset by identifying and addressing cognitive distortions, while practising acceptance, control, and coping strategies. Relaxation techniques were incorporated to support stress reduction (Clark et al., 1995; Ellis, 2023; Thompson, 1991). In the third week, This phase emphasised the development of resilience through cognitive reframing, neuroplasticity, self-regulation, and strategies for countering irrational thoughts (Bandura, 1977; Vohs & Baumeister, 2013). The fourth week focused on the following: The final week focused on strengthening interpersonal relationships and cultivating empathy, compassion, and gratitude as psychological resources. Additional discussions covered self-efficacy and strategies for managing interpersonal challenges (Gilbert & Procter, 2006; Killen & Macaskill, 2015; Zessin et al., 2015).

The overarching objective of the programme was to enhance participants’ stress perception and resilience through structured, evidence-based daily engagement.

### Measurement Tools

Participants assessed their perceived stress and resilience using two validated instruments: the Resistance Orientation–Regeneration Orientation Scale (RS-Scale; Otto & Linden, 2018) and the Perceived Stress Questionnaire (PSQ; Fliege et al., 2009).

The RS-Scale consists of 20 items divided into two subscales. The Resistance Orientation subscale (10 items) measures resilience and goal-directed behaviors, such as “*When striving for a goal, personal emotions should not be a factor.*” The Regeneration Orientation subscale (10 items) assesses self-care tendencies, including items like “*During stressful periods, recovery time is especially important.*” Participants rated each item on a 5-point Likert scale (1 = *strongly disagree* to 5 = *strongly agree*). The internal consistency of the subscales was excellent, with Cronbach’s alpha values of α = 0.93 (Resistance Orientation) and α = 0.92 (Regeneration Orientation). Although the RS-Scale comprises two subscales, only the total composite score (sum of all 20 items) was used in the statistical analyses.

To evaluate participants’ subjective perception, appraisal, and processing of stressors over the past four weeks, the German version of the PSQ was administered (Fliege et al., 2009). The short form comprises 20 items across four subscales (Worry, Tension, Joy, and Demands), rated on a 4-point Likert scale (1 = *almost never* to 4 = *most of the time*). Internal consistency was high, with a total Cronbach’s alpha of α = 0.86 and subscale values ranging from α = 0.80 to α = 0.85. High split-half reliability values were also observed. Subscale scores were computed by summing the respective item values according to the evaluation manual.

In line with the primary objective of the study, resilience—measured using the RS- Scale—was designated as the primary outcome. Perceived stress, assessed with the PSQ, was the secondary outcome, reflecting the anticipated downstream effect of increased resilience on participants’ stress perception. This distinction was pre-specified in the study design and used to guide interpretation of the intervention’s effectiveness.

### Data Analysis

Data were collected at T1, T2, and T3 using the online platform ScoSci Survey. A mixed factorial repeated measures ANOVA was performed to examine differences over time. Any violations of sphericity were addressed through the implementation of either the Greenhouse–Geisser or Huynh–Feldt adjustments, as deemed suitable. The homogeneity of variance was tested using Levene’s test. Post hoc comparisons were conducted using Tukey’s method when homogeneity of variance was observed, and Holm’s method was employed otherwise (Chen et al., 2018; Holm & Christman, 1985). The significance level was set at 0.05, based on a priori power analysis.

## Results

A total of 120 participants were randomly assigned in equal numbers to the experimental group (*n* = 60) and the waitlist waitlist control group (*n* = 60). The sample consisted of 65% women (*n* = 78) and 35% men (*n* = 42), with a mean age of 41.4 years (*SD* = 12.5).

### Resilience (RS-Scale)

A mixed ANOVA revealed a significant time × group interaction for overall resilience scores (total RS-Scale composite), F(1.18, 139.39) = 51.45, *p* < .001, partial η^2^ = .30.. Post hoc comparisons indicated significant improvements in the experimental group from baseline (T1) to post-intervention (T2), with a mean difference of 4.4 (*p* < .001, *d* = 0.4), and from T1 to three-month follow-up (T3), with a mean difference of 7.52 (*p* < .001, *d* = 0.6). In contrast, the waitlist control group did not exhibit significant changes across any time points.

**Fig. 1:**
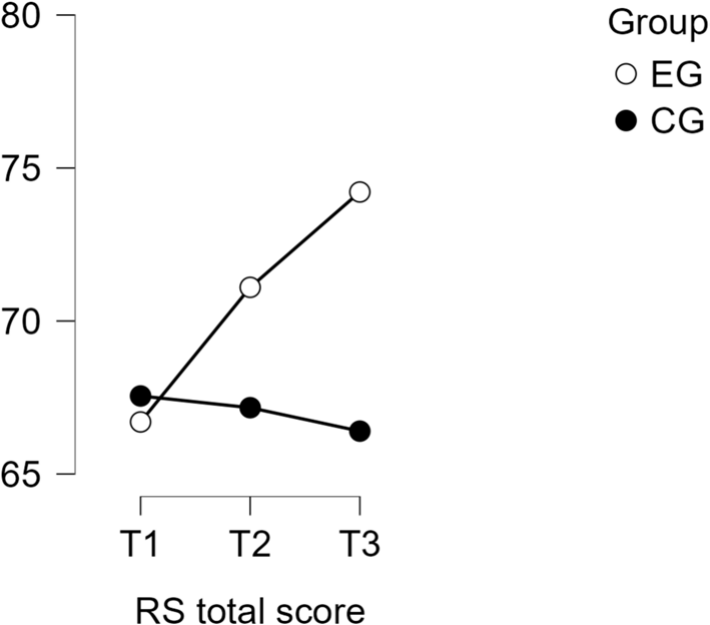
Progression of resilience scores across time points. (Note: Mean resilience scores (RS) for the experimental and waitlist control groups at T1 (baseline), T2 (post-intervention), and T3 (three-month follow-up). Higher scores indicate greater resilience.)

At T3, the difference in resilience scores between the experimental and waitlist control groups was statistically significant, with a mean difference of 7.82 (*p* = .002, *d* = 0.7), indicating a medium effect size favoring the experimental group. A summary of resilience scores across time points is provided in Table 1, and the progression is visualized in Fig. 1.

**Table 1:**
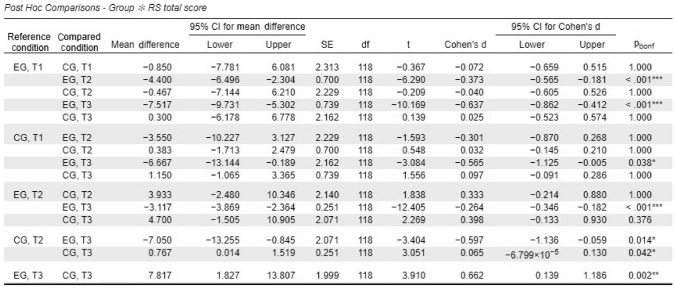
A summary of resistance orientation–regeneration orientation scale total score results from T1 to T3 for Experimental (EG) and Control (CG) Groups

*p < .05, **p < .01, ***p < .001

*p*-value and confidence intervals adjusted for comparing a family of 15 estimates (confidence intervals corrected using the bonferroni method)

*EG* experimental group; *CG* control group. T1 = baseline; T2 = post-intervention; T3 = 3-month follow-up

### Perceived Stress (PSQ)

For the PSQ total score, a significant time × group interaction was observed, *F*(1.05, 123.56) = 29.38, *p* < .001, partial η^2^ = .20. The experimental group showed significant reductions in perceived stress from T1 to T2, with a mean difference of 13.67 (*p* < .001, *d* = − 0.9), and from T1 to T3, with a mean difference of 22.06 (*p* < .001, *d* = − 1.4). In contrast, the waitlist control group exhibited no significant changes across any time points. At T3, the total stress score in the experimental group was significantly lower than in the waitlist control group, with a mean difference of − 19.58 (*p* < .001, *d* = − 1.23), representing a large effect size. Significant time × group interactions were observed for all PSQ subscales:

**Worry subscale**: *F*(1.12, 124.33) = 25.67, *p* < .001, partial η^2^ = .18.

**Tension subscale**: *F*(1.08, 121.22) = 19.34, *p* < .001, partial η^2^ = .16.

**Joy subscale** (inverted for *lack of joy*): *F*(1.09, 123.01) = 21.02, *p* < .001, partial η^2^ = .17.

**Demands subscale**: *F*(1.04, 120.54) = 18.77, *p* < .001, partial η^2^ = .15.

**Fig. 2:**
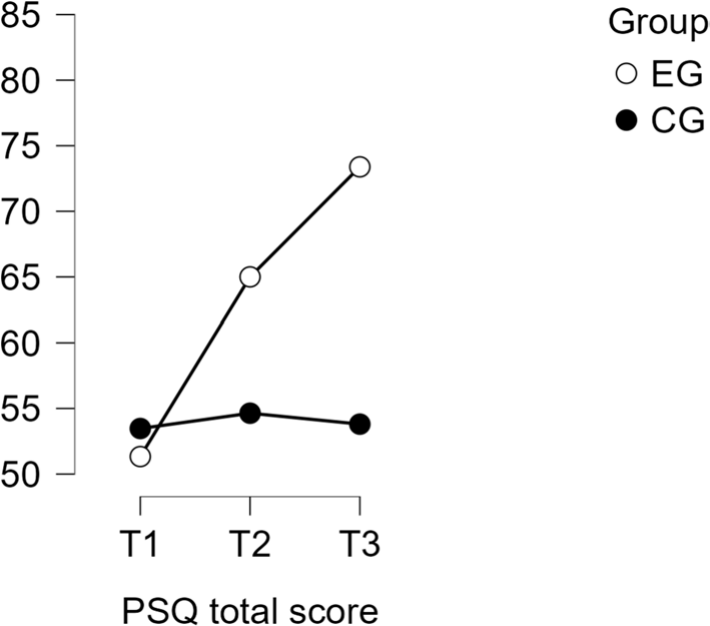
Progression of perceived stress scores (PSQ total) across time points. (Note: Mean PSQ total scores for the experimental and waitlist control groups at T1, T2, and T3. Higher scores reflect decreased stress perception.)

Post hoc analyses revealed significant improvements in all PSQ subscale scores in the experimental group between T1 and T3, with large effect sizes observed across subscales. The waitlist control group, however, did not show significant changes for any subscale. A summary of these results is presented in Table 2, while Table 3 provides detailed statistical outcomes for the subscales. The progression of PSQ total scores across time points is visualized in Fig. 2.

**Table 2:**
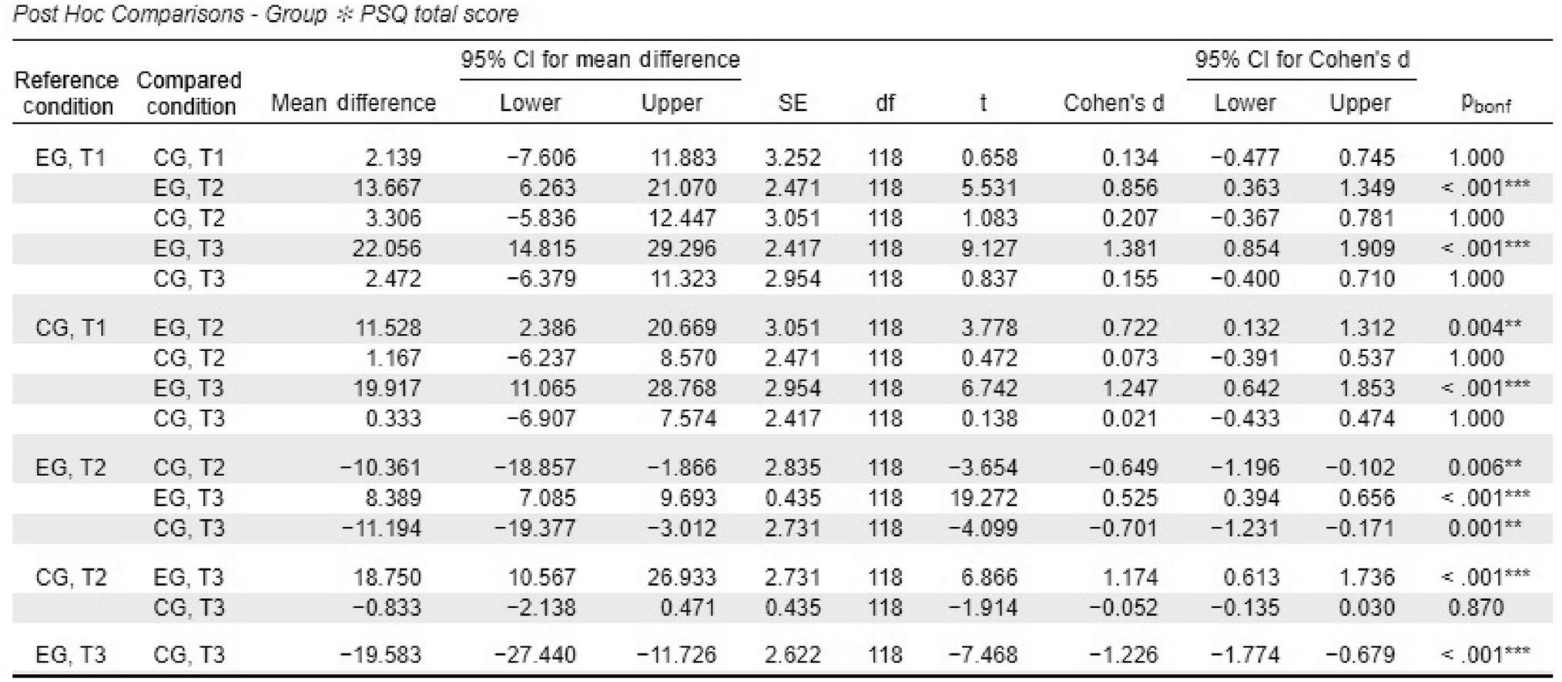
Summary of perceived stress questionnaire subscale results from T1 to T3 for experimental (EG) and control (CG) groups

**p < .01, ***p < .001

*p*-value and confidence intervals adjusted for comparing a family of 15 estimates (confidence intervals corrected using the bonferroni method)

*EG* experimental group; *CG* control group. T1 = baseline; T2 = post-intervention; T3 = 3-month follow-up

**Table 3:** Mixed ANOVAs for perceived stress questionnaire (PSQ) subscales, T1 to T3

PSQ subscale	F-value	DF	*p*-value	Partial η^2^	Post-Hoc *p*-value	Mean difference (MDiff)	95% confidence interval (CI)	Cohen’s d (effect size)
Worries	16.75	1.17, 137.67	< .001	.12	< .001	− 17.44	[− 26.62, − 8.27]	− 0.9
Tension	25.63	1.10, 130.25	< .001	.18	< .001	− 23.33	[− 32.52, − 14.15]	− 1.2
Joy	13.87	1.13, 133.03	< .001	.11	< .001	16.33	[6.74, 25.93]	0.9
Requirements	19.06	1.14, 134.85	< .001	.14	< .001	− 21.22	[− 30.56, − 11.89]	− 1.1

Post hoc comparisons reflect between-group differences at T3. Negative mean differences indicate lower scores in the experimental group relative to controls

## Discussion

The results of this randomized controlled trial demonstrate that a 28-day digital resilience training programme significantly improved resilience and reduced stress in nursing and direct care staff. Participants in the experimental group achieved higher RS-Scale scores post-intervention and exhibited a significant reduction in stress levels, particularly in the ‘worry’ and ‘tension’ sub-dimensions of the PSQ scale.

Our results align with previous findings on resilience training effectiveness. For example, Kunzler et al. (2020) demonstrated that resilience training reduced stress and depression symptoms in healthcare professionals. Additionally, our findings highlight the efficacy of a fully digital intervention, addressing organizational and scheduling barriers of traditional face-to-face programmes (e.g., Henshall et al., 2023). Another important finding is the strong reduction in perceived stress, consistent with Zhai et al. (2021), who reported significant improvements in resilience, stress, and burnout. In particular, our PSQ results reinforce the link between resilience and stress regulation (Fliege et al., 2009). Unlike synchronous group-based programmes (e.g., REsOluTioN), our study demonstrated that an individualized, asynchronous approach can yield comparable or superior results. A notable comparison is Janzarik et al. (2022), whose group-based therapy facilitated in-depth stressor processing but lacked flexibility for shift workers. In contrast, our programme offers a more accessible and time-flexible alternative, making it particularly suitable for nursing and direct care staff. These findings are highly relevant globally, as resilience promotion is increasingly recognised as a key strategy for addressing mental health challenges in the nursing profession (cf. Henshall et al., 2023; Kunzler et al., 2020). Interestingly, while both outcomes showed significant improvements, the reduction in perceived stress yielded a larger effect size than the increase in resilience. This suggests that digital training may produce measurable emotional relief even within a short timeframe, while the development of deeper resilience capacities may require sustained engagement.

Our results underscore the importance of scalable interventions tailored to the realities of patient care staff. High workloads, irregular shifts, and staff shortages often impede participation in face-to-face training. Here, the tested digital resilience programme provides a viable and adaptable solution. The 100% completion rate suggests digital interventions are well received when tailored to the target group’s needs. Furthermore, this study demonstrated that fostering resilience enhanced mental health. Consistent with Walpita and Arambepola (2020), who linked resilience to improved job performance, our findings suggest digital training can serve as a practical intervention.

While the programme was designed for daily engagement over four weeks, we recognise that this level of consistency may be difficult to maintain in real-world clinical settings. However, the asynchronous, self-paced structure allowed participants to flexibly adjust their use of the materials—such as catching up after missed days or clustering sessions on days off. Although adherence was not tracked session-by-session, the 100% retention rate at all three measurement points suggests that the overall structure was manageable for this population. Informal feedback received from several participants indicated appreciation for the short, modular format and the option to engage during breaks or at home, although some reported challenges with daily consistency during peak work periods. Future studies may benefit from incorporating structured adherence tracking and participant burden ratings to further evaluate feasibility in routine settings.

### Limitations

Despite these promising results, some limitations must be acknowledged. First, self-reported data may be subject to social desirability bias. Second, the sample consists solely of German nursing and direct care staff, limiting generalizability to other countries and healthcare systems. Third, the absence of an active comparison intervention leaves uncertainty regarding its relative effectiveness. Future studies should compare alternative resilience-building interventions and examine long-term effects beyond three months.

## Conclusion

These findings highlight the importance of integrating resilience training into HR strategies in healthcare. Digital programmes, such as the one tested here, provide an effective way to support nursing and direct care staff’s mental health without adding workload burdens. Their flexible structure ensures accessibility for staff with non-traditional hours. A key strength of digital resilience training is its scalability, enabling implementation in underserved regions and resource-limited settings. Furthermore, such interventions may improve workforce retention, making the profession more attractive—a critical factor amid the global nursing and direct care staff shortage (Kraft & Drossel, 2019). While perceived stress showed the largest effect size, resilience—designated as the primary outcome—remains the core construct targeted by the intervention. This outcome hierarchy reflects the programme’s theoretical foundation and supports its intended long-term impact: enhancing internal coping capacities to sustainably buffer workplace stress.

Future research should assess the long-term sustainability of digital resilience training beyond 6–12 months. Additionally, evaluating its effectiveness across different cultural and professional contexts would enhance generalizability. Further refinements, such as interactive elements, personalized feedback, or gamification, could boost engagement and effectiveness.

## Data Availability

Availability of data and materials: The datasets used and/or analysed during the current study are available from the corresponding author on reasonable request.
